# *QuickStats:* Percentage* of Adults^†^ Aged ≥18 Years with Current Hepatitis C Virus Infection,^§^ by Health Insurance Coverage^¶^ — National Health and Nutrition Examination Survey, United States, January 2017–March 2020

**DOI:** 10.15585/mmwr.mm7132a4

**Published:** 2022-08-12

**Figure:**
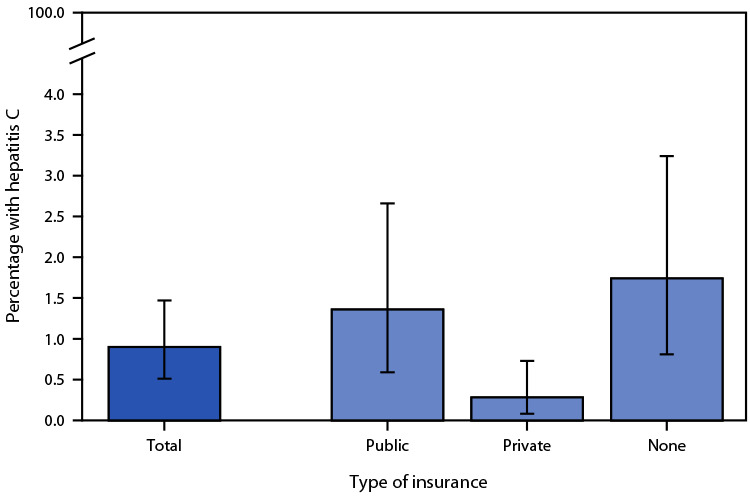
During January 2017–March 2020, an estimated 0.9% of U.S. adults aged ≥18 years had current hepatitis C virus infection. The percentage of adults with current hepatitis C virus infection was greater among those with no insurance (1.7%) or public insurance (1.4%), compared with those with private insurance (0.3%).

**Source:** National Health and Nutrition Examination Survey, January 2017–March 2020. https://wwwn.cdc.gov/nchs/nhanes/continuousnhanes/default.aspx?cycle=2017-2020

